# Animal Welfare and Livestock Supply Chain Sustainability Under the COVID-19 Outbreak: An Overview

**DOI:** 10.3389/fvets.2020.582528

**Published:** 2020-10-15

**Authors:** Nesrein M. Hashem, Antonio González-Bulnes, Alfonso J. Rodriguez-Morales

**Affiliations:** ^1^Department of Animal and Fish Production, Faculty of Agriculture (El-Shatby), Alexandria University, Alexandria, Egypt; ^2^Departamento de Reproducción Animal, Instituto Nacional de Investigación y Tecnología Agraria y Alimentaria (INIA), Madrid, Spain; ^3^Facultad de Veterinaria, Universidad Complutense de Madrid, Madrid, Spain; ^4^Public Health and Infection Research Group, Faculty of Health Sciences, Universidad Tecnologica de Pereira, Pereira, Colombia; ^5^Grupo de Investigacion Biomedicina, Faculty of Medicine, Fundacion Universitaria Autonoma de las Americas, Pereira, Colombia

**Keywords:** SARS-CoV-2, pandemic, animal welfare, livestock farming, food security

## Abstract

The COVID-19 pandemic, caused by the emergence of a new strain of coronavirus (SARS-CoV-2) around the end of December 2019, has caused a worldwide public health emergency and a socioeconomic crisis during 2020. The lockdown imposed to cope with the health issues caused by the outbreak of the disease has dramatically challenged and negatively affected all the economic sectors of the modern global economy. Specifically, the livestock sector and its related industries are among the most impacted sectors. This is mainly ascribed to the limitations of animal movement and the decrease of production inputs' availability. Other factors negatively affecting the sustainability of the livestock sector have been the shortage of workers due to the lockdown/curfew, the strong decrease in the purchasing power of the consumer, and the intensification of health care tasks. Such an impact is not only highly relevant because of their economic consequences, but also because of the effects of the lockdown and sanitary rules on animal care and welfare. The current review aims to offer: (a) a comprehensive overview of the impact of COVID-19 on the welfare of farm animals and on the performance of livestock farming systems, on food chain sustainability, and finally, on the global economy and food security; and (b) a prospective outline of alleviation actions.

## Introduction

By the end of December 2019, the world awoke to a public health crisis due to the emergence of a new strain of coronavirus, which was later named Severe Acute Respiratory Syndrome coronavirus 2 (SARS-CoV-2 or COVID-19) (1). Within a few months, this virus has spread worldwide from its origin in Wuhan (China) to cause more than 11.9 million infected cases in 188 countries and more than 545,000 deaths (2). The rapid COVID-19 outbreak drives the application, in many countries, of rigorous sanitary measures as an attempt to control the spread of the virus, including quarantines and social distancing, lockdowns, closure of transportation means, travel bans and border control, restriction on import/export activity, and the shutting down of many industrial/agricultural activities. These sudden changes in the socioeconomic status have caused a severe disruption of social and human activities and the global economy, which in turn increased the risk of food insecurity. Although it is still too early to draw a full view on COVID-19's impact on short- and long-term food security and the global economy, some effects are already being seen (3).

Globally, the COVID-19 pandemic has had a direct impact on food systems through changing the food supply-demand system, and an indirect impact through decreasing purchasing power and the capacity of food distribution and marketing, and increasing healthcare tasks (4). Economic forecasts project a 0.5% fall in global economic growth in 2020 (2.4% in 2020 vs. 2.9% in 2019) as a result of disruptions to many production and industrial supply chains (5). All these elements will evoke differentiated impacts, putting many people at risk of poverty and food insecurity (3). As a result, many economic activities and industrial sectors are, and will be, negatively affected by the COVID-19 outbreak, of which the livestock sector and related industries are among the most impacted (4, 6).

Such a fact will cause severe effects worldwide, with larger effects seen on both the economies and sociodemographics of developing areas. The livestock sector is estimated to account for about 40% of global agricultural output value and two-thirds of the 600 million livestock keepers worldwide are women, so the current situation compromises their economic empowerment (7, 8). Farm animals, at all the different livestock farming systems, play an essential role in maintaining food security and meeting socioeconomic needs (farmer livelihoods and income) (Table 1). Globally, farm animals contribute about 13% of calories and 28% of protein demands directly by providing meat, milk, and eggs, in addition to their contribution to crop production through conferring transport and manure (12). The lockdown and other restrictive actions taken to control the outbreak of the COVID-19 pandemic have negatively impacted the livestock sector, particularly the dairy and meat industries, and related processes (13, 14). There were difficulties for moving live animals and animal products (milk, meat, and eggs) to markets, limitations for seasonal border crossings (transhumance) with ruminants, reductions in the purchasing power of production logistics, and shortages of labor and professional services (15). These obstacles have led to substantial disruptions in the livestock supply chain, decreasing the economic and productive efficiency of the livestock industry (15). The Food and Agriculture Organization (16) has recently published (June 11, 2020) the latest global Food Outlook for 2020. The forecast estimates that the global total meat production (including bovine, ovine, swine, and poultry) is expected to fall by 1.7% (338.9 million tons in 2019 vs. 333.0 million tons in 2020) due to animal diseases and market disruptions. The most impacted sector within meat production is the pork production sector, with an expected drop of around 8.0% (109.8 million tons in 2019 vs. 101.0 million tons in 2020), while the beef production sector is forecast to drop around 1% (72.6 million tons in 2019 vs. 72.0 million tons in 2020). Such a dramatic drop in the pork production sector is mainly driven by the ongoing African swine fever (ASF) situation in Asia and its negative impacts on pig production and trade, due to the suspension of ASF control programs following the COVID-19 outbreak (16). Also, international meat prices have dropped by 8.6%, and the sharpest drop has been observed for ovine meat, followed by poultry, pork, and beef (16–18).

**Table 1:** State and change (absolute change, AB and relative change, RC) of the livestock sector before the COVID-19 outbreak shows global animal population (1961–2014), global and regional meat and milk productions (1961–2018), and share of livestock sector in human nutrition (1961–2013).

**State**	**Period**		**Total change**	
**Global animal population**^**a**^ **(millions)**	**1961**	**2014**	**AC (million tons)**	**RC (%)**
Cattle	942.18	1470.0	+532.35	+57
Buffalo	88.32	194.46	+106.14	+120
Sheep	994.27	1200.0	+201.36	+20
Goats	48.73	1010.0	+662.53	+190
Pigs	406.18	985.67	+579.49	+143
Poultry	8.95	127.31	+118.36	+1,323
**Meat production**^**b**^ **(million tons)**	**1961**	**2018**	**AC (million tons)**	**RC (%)**
Africa	3.91	20.17	+16.26	+416
Asia	9.05	143.71	+134.66	+1,489
Europe	30.00	63.85	+33.84	+113
Americas	26.10	108.01	+81.90	+314
South America	6.52	46.12	+39.60	+608
Australia and New Zealand	2.14	6.11	+3.97	+185
Global production	71.36	342.42	+271.06	+380
**Milk production**^**b**^ **(million tons)**	**1961**	**2018**	**AC (million tons)**	**RC (%)**
Africa	11.01	46.65	+35.65	+324
Asia	42.76	352.78	+310.02	+725
Europe	194.98	226.53	+31.54	+16
Americas	83.92	185.17	+101.26	+121
South America	14.36	61.17	+46.81	+326
Australia & New Zealand	11.49	30.68	+19.19	+167
Global production	344.18	841.84	+497.66	+145
**Livestock share in human nutrition**^**c**^	**1916**	**2013**	**AC (million tons)**	**RC (%)**
Global meat consumption (kg/capita/year)	23.08	43.22	+20.14	+87
Global milk consumption (kg/capita/year)	75.55	90.00	+14.45	+19
Animal protein share (g/capita/year)	19.66	32.13	+12.47	+63
Plant protein share (g/capita/year)	41.80	49.10	+7.30	+17

a *Source: (9)*.

b *Source: (10)*.

c *Source: (11)*.

The closure of meat and milk processing plants has also contributed to the sharp disruption in the livestock supply chain, leading to substantial losses in production capacity and animal products. For instance, in the USA, the loss of production capacity due to the closure of plants have reached up to 25 and 43% for beef slaughterhouses, respectively (19). The pork industry was more affected by the crisis, with more than 10 million hogs removed from the supply chain between April and September 2020. This equates to a reduction of more than 2 billion pounds of pork in the marketplace, or more than 7% compared with total production in 2019 (19). Concomitantly, the economic losses caused by COVID-19 in the beef industry are expected to reach $13,617,418,450 (20). The same applies to the milk industry, which expressed huge losses as thousands of fresh milk gallons were dumped worldwide (21). The milk industry was also hit by low milk prices, which declined by 4.6% on average when estimated for 70 countries, and even reached 29 and 19% in the USA and India, respectively (22).

This review discusses how the COVID-19 outbreak may affect animal welfare and livestock, the dairy and meat industry, and supply chains by using available scientific/organizational reports, and cited observations and complaints of farmers. Such data may help in taking suitable measures to improve the current status and to pose lessons for the future, avoiding the emergence of food insecurity.

## Coronaviruses Transmission Through Farm Animals

Sixty percent of emerging contagious diseases have a zoonotic origin. Coronaviruses (CoVs) were first identified in the mid-1960s and are known to infect humans (*Homo sapiens*) and other animals, including birds and mammals (1). CoVs are single-strand RNA viruses that mainly target epithelial cells in the respiratory system and alimentary tract. Some of these viruses, such as infectious bronchitis virus (IBV), swine enteric CoVs, and bovine coronavirus (BCoV), can cause diseases that have a significant impact on the livestock industry. Other CoVs, such as ferret systemic coronavirus (FRSCV), feline infectious peritonitis virus (FIPV), and mouse hepatitis virus (MHV), target companion (ferrets, *Mustelaputorius furo*, and cats, *Feliscatus*) and laboratory (mice, genus *Mus*) animals (Table 2). In farm animals, these viruses can induce significant negative impacts on farm animals' health, well-being, and productivity. As an example, BCoV affects cattle and other livestock species, including horses (*Equus ferus caballus*) and camels (genus *Camelus*), inducing diarrhea, fever, and respiratory diseases, and thus causes negative impacts on animal welfare/productivity and farm profitability (1, 27).

**Table 2:** Examples of coronavirus (CoVs) strains and accompanying symptoms in different animal species.

**Virus synonym (CoVs)**	**Genus^**a**^**	**Host**	**Symptoms**	**References**
Sever acute respiratory syndrome coronavirus-2 (SARS-CoV-2 or COVID-19)	Betacoronavirus	Humans rodents, civets, cats, pangolins, minks, dogs and cats	Fever, dry cough, tiredness, shortness of breath, sore throat, headache, diarrhea, and vomiting.	(23–26)
Swine enteric CoVs: • Transmissible gastroenteritis virus (TGEV). • Porcine epidemic diarrhea virus (PEDV). • (c) Swine acute diarrhea syndrome coronavirus (SADS-CoV).	Alphacoronavirus	Swine	• Diarrhea, vomiting, rapid weight loss, high mortality in young pigs. • Same TGEV symptoms but with lower spreading and mortality rates. • Acute enteritis and mortality.	(22, 27, 28)
Swine enteric CoVs: • Porcine hemagglutinating encephalomyelitis virus (PHEV)	Betacoronavirus	Swine	• Subclinical neurotropic symptoms and high mortality and morbidity rates in young piglets (<4 weeks of age).	
Swine enteric CoVs: • Porcine deltacoronavirus (PDCoV).	Deltacoronavirus	Swine	• Clinical severity of PDCoV is mild than other porcine CoVs.	
Bovine coronavirus (BCoV)	Betacoronavirus	Bovine species (cattle), horses and camels	Bloody diarrhea, respiratory form of shipping fever, and high mortality rate.	(1, 27, 29)
Infectious Bronchitis Virus (IBV)	Gammacoronavirus	Avian/Poultry	Respiratory illness, urinary tract infection, reproductive disturbances, and high mortality and morbidity rates.	(1, 27)
Ferret systemic coronavirus (FRSCV)	Alphacoronavirus	Ferret	Anorexia, weight loss, diarrhea, and hypergammaglobulinemia.	(28)
Feline infectious peritonitis virus (FIPV)	Alphacoronavirus	Feline	Diarrhea and mild or upper respiratory signs.	(25)
Mouse hepatitis virus (MHV)	Betacoronavirus	Mice, ferrets and cats	Respiratory illness, neurotropic symptoms, and damages in vascular endothelium, hemopoietic tissue and liver.	(24)

a *Genome size range of Alphacoronavirus, Betacoronavirus, Gammacoronavirus, and Deltacoronavirus is 27–29, 26–32, 27–32, and 26–26.5 kb, respectively*.

The newly identified COVID-19 is a member of the order *Nidovirales*, family *Coronaviridae*, sub-family *Orthocoronavirinae*, genera *Betacoronavirus*, and subgenus *Sarbecovirus* (29). The invasion of this virus to the host cells depends on the ability of glycoprotein spike proteins on its envelope surface to bind with specific receptors in host cells, namely angiotensin-converting enzyme 2 (ACE2) receptors, and can affect humans and some animals (30). After the first detection of this virus in humans and the emergence of the terrible pandemic events, many studies have been carried out to identify its origin, modes of transmission, and jumping potency among species. Investigations revealed that wild animals, such as bats (*Rhinolophus* species), rodents (order *Rodentia*), civets (*Civettictis civetta*), cats, pangolins (Family *Manidea*), minks (*Neovisonvison*), and tigers (*Panther atigris*), were found to test positive for COVID-19 (1). Also, pets living closely with COVID-19-infected households, such as dogs (*Canis lupus familiaris*) and cats (23), and farmed animals, such as minks (24), have been shown to be susceptible to the virus infection. On the other hand, a serological survey that tested pigs (*Sus scrofa*), cattle and sheep (Family *Bovidae*), horses, and poultry for antibodies of COVID-19 revealed negative results (25, 26).

The overall findings of these studies led to the conclusion that: (1) the main reservoir of COVID-19 are bats; (2) some wild (bats, tiger, and civets), farmed (mink), and domestic (cats and dogs) animals can be a reservoir; (3) there is a possibility for the transmission of COVID-19 from humans to animals, animals to humans, and human to human; and (4) livestock belonging to the family *Bovidae* (cattle and small ruminants) or *Suidae* (pigs) as well as poultry are not COVID-19 reservoirs (1, 28). Despite the range of evidence suggesting that farm animals are not COVID-19 reservoir/hosts, caution should be taken regarding farm-animals-humans contact. Lessons from previous pandemics related to farm animals, and the ability of CoVs to express genetic mutations and modifications allowing them to jump among species, highlight the possibility of farm animals to be one of the COVID-19 hosts. For instance, the swine acute diarrhea syndrome coronavirus (SADS-CoV) isolated from piglets had a 95% genomic identity with horseshoe bat (*Rhinolophus* species) coronavirus HKU2, suggesting both a bat origin of the pig virus and a jumping across species (31, 32). Another example of the ability of CoVs to change among animal species or from animals to humans is the transformation of human coronavirus-229E and -OC43 (HCoV-229E and HCoV-OC43) in their intermediate hosts', alpacas and cattle, respectively before infecting final human hosts (29). Accordingly, some virologists did not exclude farm animals from the list of expected hosts for the new COVID-19 virus (29, 31). Hence, due to the possible role played by farm and wild animals in SARS-CoV-2 (COVID-19) infection, the World Health Organization (WHO) recommended the avoidance of unprotected contact with both farm and wild animals in their recent coronavirus (COVID-19) situation report (31).

Regarding the handling and consumption of animal products, there is no direct evidence on food-borne infection, including meat and milk. Precautions and hygienic measures are advisable since there is a possibility that contamination of food materials can occur through the handling of food by infected individuals. These precautions and sanitary measures include hand washing or sanitizing following the processing of fresh and packaged animal products, avoiding contact between fresh and cooked food materials, consuming well-done cooked meat, and extending meat and meat-processed materials' freezing time by more than 2 days before consumption (14).

## Impacts of COVID-19 on the Welfare of Farm Animals and Livestock Farming Systems

It is expected that the sudden restriction on human activities and the economic crisis will affect farming and veterinary services, and therefore, affect animal health (6). The COVID-19 outbreak negatively disrupted activities related to livestock welfare. The sudden restrictions on the activities of farmers, workers, and veterinary professionals led to insufficient applications of daily routine farming work. Such a situation limits a close monitoring of animal requirements and health status and thus impedes the right intervention to tackle any rising problems. Under such conditions, many farmers have taken to overstocking their animals, which increases crowding related-stress and devastates the immune system functions. Thus, the risk of animal disease prevalence is highly increased, affecting the welfare and productivity of stocking animals (30). Some farmers have had to cull their animals or to apply measures which conflict with animal welfare, such as inducing abortion and slaughtering, to decrease the population of animals inside their farms and to limit the excess production of animal products (meat and milk). Some pig farmers have been forced to abort their animals due to the short breeding-marketing cycle of these animals and the fall of consumers' demand (33). Some farmers have used cruel methods to kill thousands of pigs, such as the ventilation shutdown method. This method depends on shutting down the ventilation sources with a rise in the temperature of barns, which decreases oxygen levels and thus suffocation (34, 35).

Furthermore, some associations, such as the Canadian Cattlemen's Association, has called farmers to put cattle on maintenance rations rather than finishing rations to limit feedlot placements, coping with the closure of processing plants, and limited market capacities (36). Also, the shortage of available feed resources due to the limitations of diet ingredients (mainly grains and seeds, such as soybean and corn) and the restriction on animal movements in pastures drive some farmers to keep animals on a marginal maintenance plan of nutrition or to use alternative feed resources, which represents another animal welfare conflict (37). Indeed, many farmers failed to obtain essential veterinary services, including routine health check-ins, drugs, vaccines, and testing and diagnostic tools. Thus, disease control of animals has become an additional challenge conflicting with animal welfare aspects. Unfortunately, the effect of COVID-19 on the activities related to animal healthcare is not restricted only on the farm-scale, but there are also national and international restrictions. Many national and international animal health care programs/projects are post-poned and suspended due to budget restrictions, which limits the success of controlling disease eradication (15). In a recent commentary, Gortázar and de la Fuente (6) indicated that the COVID-19 outbreak would drive negative impacts on the control of diseases that are already present in Europe, mainly ASF. This forecast was ascribed to the possibility of increased wildlife-livestock contacts due to human confinement, and thus increased numbers of wildlife reservoirs (wild pigs), disruption of ongoing testing schemes for endemic diseases, and lower disease surveillance efforts. Overall, funds and programs directed to control outbreaks of many current transboundary animal diseases, such as ASF, foot and mouth disease, avian influenza, and other infectious animal diseases, have been severely compromised worldwide (38, 39). These events are caused by indirect effects of the COVID-19 pandemic on livestock health and productivity, as seen with other zoonotic diseases (39).

## Impact of COVID-19 on Food Chain Sustainability

**Figure 1:**
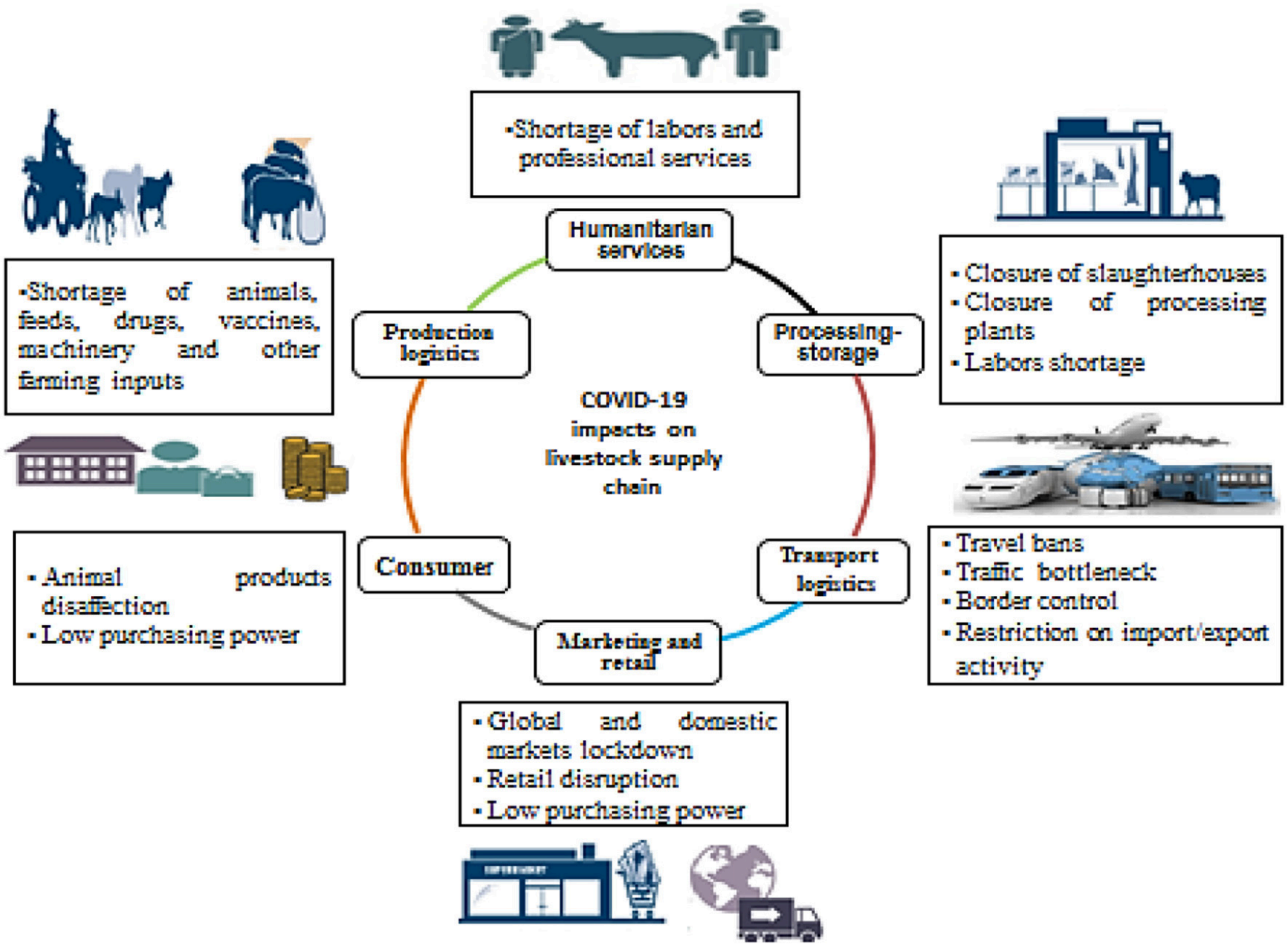
Impacts of the COVID-19 pandemic on the livestock supply chain.

Livestock production farming systems and their supply chains are challenged by many logistical supply interruptions, like most other agricultural enterprises. The COVID-19 outbreak interrupted livestock chain sustainability in different areas, from the production process to marketing and consumption of animal products (Figure 1). The primary production logistical interrupter was the shortage of accessing farming inputs, such as animal feed resources (23), livestock movements for pasture and water, and animal equipment, such as milking machines, vaccines, and other pivotal production inputs. Indeed, calls to stay at home and social distance have affected the humanitarian-dependent services in farms, affecting the routine work and animal husbandry (low number of laborers, veterinarian visits and services, and workers in product processing). Additionally, processing of animal products, such as milk and meat (delivery failure and decreasing processing and slaughtering capacities), presents another obstacle to the completion of the production cycle, forcing farmers to reduce production capacity and waste products (6). The pandemic's impact on the livestock supply chain continue to affect local and global marketing process (reduced marketing opportunities, block of import/export activity, and lower purchasing power) and consumers', demand (misconception regarding animal products safety and reduced consumers' income). All of these interruptions in the livestock supply chain put the producers (farmers) at risk of not being able to continue in the field. This situation has dramatically threatened the sustainability of livestock production systems and global food security, particularly animal protein resources (40).

### Production Facilities and Farming Inputs

Restrictions on import/export activities and local movements have prevented farmers from accessing livestock production inputs and given them limited marketing opportunities. The limitation of movements and the disruption of national and international trade routes drive to a substantial decrease in vital livestock farming materials and facilities, such as feed materials, replacement stocks (e.g., day-old chicks, piglets, gilts, heifers, and semen straws), drugs and vaccines, feed additives, and other livestock farming inputs (41). For instance, Argentina, the world's biggest soybean meal exporter, has reduced its exportation by about 50% to feed manufacturing factories, which could affect the availability of one of the most critical feed ingredients in the diets of farm animals (13). A similar situation has occurred in Brazil and the USA, where the COVID-19 pandemic restricted both soybean and corn exports, leading to a shortage of animal diet ingredients (13, 40). Movement restrictions have not only limited international trade activity, but have also constrained access to national/local production inputs; as an example, pastoralists in Africa's drylands, who depend on natural plants in pastures to feed their animals, have lost this vital natural production input and therefore pastoralist livelihoods (40).

Several companies working on vaccines, animal health products, feed additives, milk testing kits, and spare parts of pasteurization equipment to small-scale meat and dairy producers have indicated that the reductions in fluidity and foreign exchange were other significant factors affecting the sustainability of the livestock supply chain, specifically in developing countries (42). There is no doubt that such reductions in the production inputs and their trade can easily hamper the productivity of animals, the profitability of livestock producers' (specifically small-scale meat and dairy holders), and the profitability and commercial sustainability of these companies themselves.

### Human Activity and Workforce

It is widely believed that agricultural sectors, including meat and dairy production sectors, are heavily labor-dependent sectors. The COVID-19 outbreak has caused a severe shortage of labor numbers and workforce in both developed and developing countries, which is more problematic for countries that depend on an imported workforce, such as European countries (43). This shortage of domestic and seasonal and migrant laborers was ascribed to several factors. Many laborers/workers have been infected and isolated in quarantine, while some have not had the ability to pass across borders due to border control and suspension of visas (44, 45). Some laborers have left their work due to social aspects, such as family commitments or avoidance of contagion (37, 46). In France, staff shortages ascribed to the spillover of infection among stuff, quarantine, and childcare have reached 30% in some slaughterhouses, with similar instances in Italy, Egypt, Tunisia, and Jordan (46).

The lack of laborers and human activities drive significant disruptions in the worldwide sustainability of the supply chains for livestock, meat, and milk. For example, in China, the presence of some employees under quarantine has decreased the number of operating slaughterhouses in many provinces; even the operating ones cannot work at full capacity, leading to a shortage of meat supplies and sharp increases in meat prices in local Chinese meat markets, such as the *Xinfadi* market in Beijing's largest wholesale market (47). Also, a shortage of laborers working in meat processing farms and plants was one of the primary concerns in Canada and the USA, particularly when the visa of foreign workers was suspended (44). In the USA, about 20% of pork production and 10% of beef production have been stopped due to the COVID-19 spillover among staff (37, 38). In Austria, the meat processing industry depends to a large extent (80%) on the foreign workforce (migrants from eastern European countries). Any restrictions on free movement at borders has an impact on the processing chain (48). The same applies to dairy farms, since trucking companies distributing milk products from dairy farms have suffered from a shortage of drivers, as many of them have stopped working to avoid contagion probability (21).

### Distribution and Retail

Distribution and retail are essential livestock supply chain elements, successful delivery and retail guarantee the connection between the producers and consumers, completing the production-demand chain. This part of the livestock supply chain has been dramatically affected and challenged by many logistical obstacles under the impact of the COVID-19 outbreak. One of the major impediments that have faced distribution and retail processes was related to local vehicles' movement and road traffic controls, as well as the lockdown of vast areas.

In the specific case of China, transportation bottlenecks in main roads have affected the local meat supply, and family farms cannot market hogs as trucks cannot enter villages due to the lockdown (5). In the Philippines, delays of vehicles transporting raw materials for processing meat threatened to cause a shortage until movement bans were loosened. Similarly, milk distribution was disrupted by tight road traffic controls, leading to milk dumping (46).

Also, a limited processing capacity for meat and milk products has negative impacts on the distribution and retail of animal products. The Beef Farmers of Ontario organization has asked its members to delay the sale of cull cows because the beef processing sector doesn't have the adequate capacity for processing meat products (37). In the USA and Canada, pig farmers have culled or aborted their animals due to the closure of processing facilities, which is a barrier to the completion of selling and shipping operations (33).

Another challenge is the dramatic increase in cargo shipping charges accompanied with low product prices. Some export firms have complained of the high delivery transport costs of live animals and frozen meat products (42). In context, the USA dairy farmers have suffered from substantial production costs and low prices of raw milk due to the reductions in distribution and retail of milk processing industries and processed milk products for markets (48).

### Trade and Marketing Opportunities

The COVID-19 pandemic's impact on livestock supply chain sustainability have continued to affect local and global trades and marketing opportunities. To imagine how the COVID-19 pandemic affects global trade, it is enough to know that global trade has decreased by 13–22% during the few months of the pandemic emergence (49). Many livestock producers and traders have lost their global or local market opportunities, and thus their incomes. Because the world is currently one unit, global movement restrictions have impacted trade worldwide. In Asia, movement restrictions have stopped livestock trade to China from the Lao People's Democratic Republic, Thailand, Myanmar, and Vietnam. In Latin America, especially in Argentina and Uruguay, meat export drops have reduced farmer revenues (46, 49). Similarly, in Northern America, the USA pig prices dropped by roughly 27% in just over a week as a result of limited access to markets and slaughterhouses/processing plants. Within the European Union (EU), ~4.3 million head of cattle, 3.5 million sheep and goat heads, 33.4 million pigs, and 1,000 million poultry were traded alive between EU countries in 2018. More than 1.8 million head of cattle are exchanged between Belgium, Ireland, Spain, Greece, France, and Italy (50). Transport of live animals has been affected by the reintroduction of border checks, disrupting business but also delaying times for transportation which, in turn, is against animal welfare (50).

This situation has resulted in a specific decrease in farm gate prices in some EU countries, such as Poland, where domestic consumption only represents 15% of production (46). In Denmark, the beef and veal sectors reported a significant decrease in exports to the southern part of Europe during the outbreak. The same applies to dairy sectors, as some dairy producers have lost access to export markets [e.g., Austria dairy producers and their target spot market in Italy; (48)].

The situation has become even worse in developing countries and for household breeders, who are challenged by poverty and low individual incomes. Taking East Africa as an example, many livestock producers' revenues, mainly pastoralists, rely on seasonal exports of live animals and frozen meat during specific marketing seasons, such as Ramadan, pilgrim season, and Eid to the Middle Eastern countries. Thus, movement restrictions have threatened their yearly income and livelihood (3). The impact of COVID-related movement restrictions have continued to increase the challenge of some marginalized producers, such as rural women, who are unable to access markets under the lockdown restrictions to earn their household incomes from informal trading of small livestock, dairy products, and poultry in local markets (3, 40).

In general, the COVID-19 outbreak has diminished many marketing opportunities by minimizing the demand of many routine consumers, such as schools, restaurants, local markets, hotels, the institutional sector, and the tourism industry.

### Role of Consumer and Livestock Supply Chain Sustainability

Consumers play a critical dual role in the creation and completion of any food supply chain. The consumer is the primary and final target of any food supply chain, and thus the final product of the supply chain has to satisfy consumer demand and ambition. At the same time, the continuation of the food supply chain depends on the demand and purchasing power of the final product by the consumer. Under the effect of the COVID-19 pandemic, the directions of the consumer toward consumption of livestock products, meat, and milk have been reconsidered, taking into account two main aspects: purchasing power and food staple priorities, and general awareness regarding the safety of animal products. Comparing demand for meat and dairy staples with other food staples, such as grains, sugar, roots, and tubers, reveals that the demand for meat and dairy staples is less elastic. This is mainly due to the high prices of animal product staples compared to other staples, particularly those of carbohydrate origin, which is not suitable for the consumer under the pandemic conditions as many consumers have lost their income or have a low budget (5, 40). Regardless of the impact of the purchasing power of consumers on the sustainability of livestock supply chain, consumers, misconception regarding the ability of food, including livestock products (meat and milk), to be a vehicle for the virus transmission plays a critical role in maintaining the demand for animal products, increasing demand-side shocks (51).

## Impacts on the Global Economy and Food Security

The disruption in the livestock supply chain and accompanying imbalances between production and demand sides have crucially impacted livestock sector economies. However, it is too early to draw a real overview of the size of the economic crisis that will affect livestock sector economics. Early reports have shown that the imbalance in livestock sector economics will be long-term and will continue into 2021. According to the National Cattleman's Beef Association, the cattle industry in the USA is expected to lose about $13.6 billion through 2021 as a result of the COVID-19 spillover (52). Peel et al. (20) have expected losses in the cow-calf sector by estimating weighted average damage. They expected that this sector might lose about $3.7 billion due to low sales values (low prices). In addition to the loss in calf sales value, the loss in value of the breeding stock is estimated at $4.45 billion ($142 per mature breeding female). Similarly, the hog industry is expected to lose around $5 billion (20).

A significant waste in food resources, specifically meat and milk, was one of the consequences of the COVID-19 outbreak. For instance, dairy farmers have been forced to throw out a million gallons of milk due to the decline in demand for milk and dairy products (53). Under the new global food security situation, such waste in food resources is a horrible further crisis in addition to the health crisis. At the beginning of 2020, there were 27 million acutely food-insecure people in 35 countries in “emergency” conditions, who could potentially be on the brink of famine due to the direct and indirect impacts of the COVID-19 pandemic. In April 2020, the Famine Early Warning Systems Network Global Food Security Alert warned of the risk that populations in developing countries (such as those in northeastern Nigeria, South Sudan, and Yemen) could be challenged by famine due to the COVID-19 pandemic (3, 54). In light of this scenario, there is an urgent need to find measures and unconventional solutions to control the negative impacts of the pandemic on global food security. Specifically, the current crisis has shown the fragility of the worldwide economy even in developed countries and the possibility of an increased risk of food insecurity.

## Current and Prospective Alleviation Measures

Facing the repercussions of the COVID-19 outbreak on the sustainability of food supply chains has become an urgent mission to guarantee global food security. Taking the livestock supply chain as an example, as shown in this overview, there are risks at each point of the supply chain threatening its sustainability. Mitigation of these risks requires specific collaboration between different entities, including governments, policymakers, non-governmental institutions/organizations, and scientists. From governmental and institutional sides, various actions have been taken to support livestock farming systems as an attempt to keep livestock supply chains functioning. These measures rely on: (1) facilitating the direct distribution of food/animal products to consumers (Brazil, Italy, Ghana, and the Philippines) and offering electronic marketing platforms (China and Morocco); (2) providing livestock production inputs, such as animal feeds, drugs, and machines [e.g., the Italian authorities have purchased ultra-high-temperature (UHT) milk from dairy farmers]; (3) supporting the resumption of meat/milk processing enterprises (China); (4) creating agricultural, healthcare, and animal diseases control extension programs; and (5) providing direct funding support to seasonal and informal labors (Egypt, Tunisia, and Morocco) (46, 49, 55).

From the scientific community side, prospective alleviation strategies that may improve the resilience of the livestock supply chain have been proposed. Introducing new protein sources, such as insects, algae, and dairy-processing by-products (e.g., whey proteins), to the food supply chain as alternatives to animal protein sources is one of the suggested strategies (14, 56). Another ambitious option relies on the application of tissue culture biotechnology to grow animal muscle *in vitro* as an eco-friendly production technique, as methane emission by farm animals will be reduced (57, 58). However, these strategies seem not sustainable enough and may need ethical and social acceptance; other alleviation strategies would be more realistic. Farm animals as the main element of this food supply chain should be more adaptable to environmental stresses, such as diseases, poor management, and non-standard well-being conditions. In this term, improving immunity and the withstanding of animals to diseases and harsh conditions, while concomitantly maintaining adequate production potentials, should be one of the practical applications. Creating animals with a more efficient immune system may indirectly contribute to decreasing the cost of production as the utilization of medications and drugs, specifically antibiotics, could therefore be minimized (59). This also will benefit consumers and human health by reducing veterinary drug residues in animal products (60, 61). Furthermore, improving the immune system functioning of animals may help in maintaining the biodiversity of farm animals by eliminating the need to cull or kill infected or suspected animals under pandemic outbreak circumstances (17). Recently, genetic improvements using advanced transgenesis and genome editing technologies, such as CRISPR-Cas, may facilitate rapid modulation of the genome of farm animals, creating animals that can efficiently adapt to the environmental and breeding challenges (62). In this term, scientists have shown the opportunity of these techniques to control many farm animals' infectious diseases, such as ASF and bovine tuberculosis (62).

The availability of animals', feed ingredients, either through foraging or concentrate ingredients, was an additional challenge confronting the sustainability of the livestock supply chain. Data presented in our overview illustrated that one of the livestock supply chain's major disruptors was the inability of farmers to access animal feed. The restriction on import/export activity has negatively impacted the availability of essential feed ingredients [soybean and corn; (13, 40)]. Accordingly, strengthening the livestock supply chain through alternative feed resources is one of the most critical measures that can improve the resilience of this food supply chain. An emphasis on harnessing local feed resources and/or introducing agro-industrial by-products as alternative feed resources may present an effective solution (63). That could be achieved by applying different feeding strategies, such as increasing the forage-to-concentrate ratio, use of straw-based feed blocks, recycling of human food waste and human-inedible food components to feed, and recycling of agricultural wastes/byproducts (64). Also, in regions where the feeding of animals depends on natural pastures, reforming of pastures by introducing permanent growing plant species may contribute to providing sustainable feed resources for grazing animals (65).

The experience acquired through the COVID-19 crisis may also contribute to optimizing the part of the livestock supply chain that includes processing, retail, and marketing through strengthening the connection between producers, retailers, targeted markets, and consumers (66). The inefficient relationship between these key elements has resulted in significant waste in animal products (milk and meat), which could be avoided through establishing online marketing platforms and internet technology services (67).

Unfortunately, to date there is no particular vaccine or therapeutic protocol to control the spread of COVID-19, and forecasts predict the continuation of this pandemic for a long time. In this scenario, livestock farmers have to be aware of different measures that they can apply themselves to maintain safe and efficient livestock farming. Successful farmers should maintain farm biosafety (4, 15), workers/laborers safety (17, 59), animal welfare standards (1, 50), production inputs access (59, 68), and finally should guarantee marketing of their products with sufficient revenue. The recommendation and measures that aid farmers to achieve these approaches are summarized in Table 3.

**Table 3:** Summary of simple management plans to avoid negative impacts of COVID-19 on livestock farming system.

**Item (References)**	**Measure(s)**
Farm biosecurity (4, 15)	• Maintain general hygiene of the premises • Clean and sanitize all machines and solid surfaces • Limit contact between wild animals (mice, dogs, birds, etc.) and farmed animals • Limit the presence of foreign visitors • Minimize joining new animals to the herd
Human health and safety (17, 59)	• Follow healthcare recommendations and social distancing instructions • Monitor and ensure the health status of workers • Isolate infected workers • Follow biosecurity instructions for safe human-animal contact
Animal health and welfare (1, 50)	• Maintain good animal husbandry (proper housing system) and management (e.g., milking hygiene). • Ensure the continuation of sanitary and prophylactic programs for the farm animals against common diseases • Use precision farming livestock tools for real time monitoring of animals • Follow animal well-being standards during all the production cycle, transportation, and marketing
Livestock supply chain (55, 68)	• Ensure the availability of different production inputs • Focus in using available and local feed resources • Try to use technology and online marketing platforms • Monitor the market demands and adjust production quantity and quality • Minimize sources of production wastes, e.g., in dairy farms using simple measures to control surplus milk production: - decrease milking frequency 2 instead of 3 - extend dry off period, 65 instead of 45 days - extend the time of calves weaning

## Conclusion

In this overview, we illustrated the significant disruptions that occurred in livestock, milk, and meat supply chains due to the outbreak of COVID-19. The COVID-19 pandemic revealed that, despite the vast advancements in knowledge and informatics technologies, we still need to create innovative applications and measures that can support the world against any future pandemic disasters. It is now clear that pandemic disasters are not only a matter of human health insecurity but can also encompass food insecurity and economic recession, increasing poverty and famines worldwide.

## Author Contributions

NH: creating the main idea and writing the first draft. AG-B and AR-M: reviewing and final preparation of the manuscript. All authors approved the content for publication.

## Conflict of Interest

The authors declare that the research was conducted in the absence of any commercial or financial relationships that could be construed as a potential conflict of interest.

## References

[1] Rodriguez-Morales AJ Bonilla-Aldana DK Tiwari R Sah R Rabaan AA Dhama K COVID-19, an emerging coronavirus infection: current scenario and recent developments-an overview. J Pure Appl Microbiol. (2020) 14:6150 10.22207/JPAM.14.1.02

[2] Coronavirus Resource Center (2020). Available online at: https://coronavirus.jhu.edu/map.html (accessed July 8, 2020).

[3] Food and Agriculture Organization (FAO) Coronavirus Disease 2019 (COVID-19). Addressing the Impacts of COVID-19 in Food Crises. (2020). Available online at: http://www.fao.org/3/ca8497en/ca8497en.pdf (accessed July 8, 2020).

[4] Poudel PB Poudel MR Gautam A Phuyal S Tiwari CK Bashyal N COVID-19 and its global impact on food and agriculture. J Biol Todays World. (2020) 9:221 10.35248/2322-3308.20.09.221

[5] Schmidhuber J Pound J Qiao B COVID-19: Channels of Transmission to Food and Agriculture. FAO (2020). Available online at: 10.4060/ca8430en (accessed June 28, 2020).

[6] Gortázar C de la Fuente J. COVID-19 is likely to impact animal health. Prev Vet Med. (2020) 180:105030. 10.1016/j.prevetmed.2020.105030 32447153 PMC7255270

[7] Thornton PK Kruska RL Henninger N Kristjanson PM Reid RS Atieno F Mapping Poverty and Livestock in the Developing World. Nairobi: International Livestock Research Institute (2002). Available online at: https://www.semanticscholar.org/paper/Mapping-poverty-and-livestock-in-the-developing-ThorntonKruska/772f4c523d5805a0f59ea872a4b868c798a1e332

[8] Salmon GR MacLeod M Claxton JR Ciamarra UP Robinson T Duncan A. Exploring the landscape of livestock ‘Facts'. Glob Food Sec. (2020) 25:100329. 10.1016/j.gfs.2019.100329 32566469 PMC7299074

[9] The UN Food and Agricultural Organization (FAO) Statistics Available online at: http://www.fao.org/faostat/en/#data/ (accessed August 5, 2020).

[10] Food and Agriculture Organization of the United Nations (FAO) (2020) Available online at: http://www.fao.org/faostat/en/?#data/ (accessed August 5, 2020).

[11] United Nations Food and Agricultural Organization (FAO) Available at: http://www.fao.org/faostat/en/#data/FBSF (accessed August 5, 2020).

[12] Food and Agriculture Organization (FAO) World Livestock 2011–Livestock in Food Security. Rome: FAO (2011). Available online at: http://www.fao.org/docrep/014/i2373e/i2373e00.htm (accessed August 8, 2020).

[13] Seleiman MF Selim S Alhammad BA Alharbi BM Juliatti FC Will novel coronavirus (Covid-19) pandemic impact agriculture, food security and animal sectors? Biosci J. (2020) 23:36 10.14393/BJ-v36n4a2020-54560

[14] Galanakis CM. The food systems in the era of the coronavirus (COVID-19) pandemic crisis. Foods. (2020) 9:523. 10.3390/foods9040523 32331259 PMC7230343

[15] Food and Agriculture Organization (FAO) Guidelines to Mitigate the Impact of the COVID-19 Pandemic on Livestock Production and Animal Health. (2020). Available online at: http://www.fao.org/3/ca9177en/CA9177EN.pdf (accessed June 28, 2020).

[16] Food and Agriculture Organization (FAO) Food Outlook–Biannual Report on Global Food Markets. Food Outlook, 1. Rome (2020). Available online at: 10.4060/ca9509en (accessed August 5, 2020).

[17] Pal M Kerorsa GB Zoonotic significance of COVID-19 and precautions related to animals during outbreak of the disease. J One Health. (2020) 8:39–43. Available online at: www.jakraya.com/journal/joh

[18] Phelps M COVID-19: African Swine Fever Response Challenge. Queensland Country Life (2020). Available online at: https://www.queenslandcountrylife.com.au/story/6715906/covid-19-creates-africanswine-fever-response-challenges/ (accessed June 28, 2020).

[19] Muth MK Read Q. Effects of COVID-19 Meat and Poultry Plant Closures on the Environment and Food Security. RTI International. Available online at: https://www.rti.org/insights/covid-19-effect-meat-supply-chain (accessed August 4, 2020).

[20] Peel DS Aherin D Blach R Burdine K Close D Hagerman A Economic Damages to the U.S. Beef Cattle Industry Due to COVID-19. National Cattleman's Beef Association (NCBA) (2020). Available online at: https://extension.okstate.edu/fact-sheets/economic-damages-to-the-u-s-beef-cattle-industry-due-to-covid-19.html (accessed June 30, 2020).

[21] Huffstutter PJ U.S. Dairy Farmers Dump Milk as Pandemic Upends Food Markets. World Economic Forum (2020). Available online at: https://www.weforum.org/agenda/2020/04/dairy-milk-pandemic-supplychains-coronavirus-covid19-pandemic/ (accessed June 28, 2020).

[22] International Farm Comparison Network (IFCN) Dairy Conference: Covid-19 Fear Is Bigger Than Its Impact. (2020). Available online at: https://www.dairyglobal.net/Market-trends/Articles/2020/6/IFCN-Dairy-Conference-discusses-covid-19-related-dairy-crisis-594288E/ (accessed August 5, 2020).

[23] Zhang Q Zhang H Huang K Yang Y Hui X Gao J. SARS-CoV-2 neutralizing serum antibodies in cats: a serological investigation. bioRxiv. (2020) 2020.04.01.021196. 10.1101/2020.04.01.02119632867625

[24] Malik YS Sircar S Bhat S Vinodhkumar OR Tiwari R Sah R Emerging coronavirus disease (COVID-19), a pandemic public health emergency with animal linkages: current status update. Preprints. (2020) 2020030343. 10.20944/preprints202003.0343.v1

[25] Deng J Jin Y Liu Y Sun J Hao L Bai J. Serological survey of SARS-CoV-2 for experimental, domestic, companion and wild animals excludes intermediate hosts of 35 different species of animals. Transbound Emerg Dis. (2020) 67:1745–9. 10.1111/tbed.13577 32303108 PMC7264586

[26] Shi J Wen Z Zhong G Yang H Wang C Huang B. Susceptibility of ferrets, cats, dogs, and other domesticated animals to SARS–coronavirus 2. Science. (2020) 29:1016–20. 10.1126/science.abb7015 32269068 PMC7164390

[27] Burimuah V Sylverken A Owusu M El-Duah P Yeboah R Lamptey J. Sero-prevalence, cross-species infection and serological determinants of prevalence of Bovine coronavirus in cattle, sheep and goats in Ghana. Vet Microbiol. (2020) 241:108544. 10.1016/j.vetmic.2019.108544 31928696 PMC7117134

[28] Tiwari R Dhama K Sharun K Iqbal Yatoo M Malik YS Singh R. COVID-19: animals, veterinary and zoonotic links. Vet Quart. (2020) 40:169–82. 10.1080/01652176.2020.1766725 32393111 PMC7755411

[29] Decaro N Lorusso A. Novel human coronavirus (SARS-CoV-2): a lesson from animal coronaviruses. Vet Microbiol. (2020) 244:108693. 10.1016/j.vetmic.2020.108693 32402329 PMC7195271

[30] Ghafouri-Fard S Noroozi R Omrani MD Branicki W Pośpiech E Sayad A. Angiotensin converting enzyme: a review on expression profile and its association with human disorders with special focus on SARS-CoV-2 infection. Vascul Pharmacol. (2020) 130:106680. 10.1016/j.vph.2020.106680 32423553 PMC7211701

[31] Dhama K Sharun K Tiwari R Sircar S Bhat S Malik YS. Coronavirus disease 2019–COVID-19. Clin Microbiol Rev. (2020) 33:e00028-20. 10.1128/CMR.00028-20 32580969 PMC7405836

[32] Cui J Li F Shi ZL. Origin and evolution of pathogenic coronaviruses. Nat Rev Microbiol. (2019) 17:181–92. 10.1038/s41579-018-0118-9 30531947 PMC7097006

[33] Vincent terBeek V McCullough C Covid-19 Crisis Hits US Pig Production Hard. Pig Progress (2020). Available online at: https://www.pigprogress.net/World-of-Pigs1/Articles/2020/4/Covid-19-crisis-hitsUS-pig-production-hard-576238E/ (accessed June 28, 2020).

[34] Jones D Suffocating Healthy Farmed Animals During Pandemic Is Not “Euthanasia”. (2020). Available online at: https://sentientmedia.org/suffocating-healthy-farmed-animals-during-pandemic-is-not-euthanasia/ (accessed August 4, 2020).

[35] Millions of US Farm Animals To Be Culled by Suffocation Drowning and Shooting Animals Farmed Environment (2020). Available online at: https://www.theguardian.com/environment/2020/may/19/millions-of-us-farm-animals-to-be-culled-by-suffocation-drowning-and-shooting-coronavirus (accessed August 4, 2020).

[36] Calgary AB COVID-19 Reduces North American Beef Processing Capacity: CCA Recommends Implementing Set-Aside Program. Canadian Cattlemen's Association (CCA) (2020). Available online at: https://www.cattle.ca/assets/Article/3fe08de92a/2020.04.06_CCA-PublicUpdate_-ProcessingCapacity_Alberta_COVID-19_FINAL.pdf (accessed June 28, 2020).

[37] Arnason R Animal Welfare Groups Use COVID-19 Against Farming. (2020). Available online at: https://www.producer.com/2020/04/animal-rights-groups-blame-covid-19-on-farming/ (accessed June 28, 2020).

[38] Nielsen SS Alvarez J Bicout D Calistri P Depner K Drewe JA. Risk assessment of African swine fever in the south-eastern countries of Europe. EFSA J. (2019) 17:e05861. 10.2903/j.efsa.2019.5861 32626162 PMC7008867

[39] Phelps M COVID-19: African Swine Fever Response Challenge. Queensland Country Life (2020). Available online at: https://www.queenslandcountrylife.com.au/story/6715906/covid-19-creates-africanswine-fever-response-challenges/ (accessed June 28, 2020).

[40] Food and Agriculture Organization (FAO) COVID-19 and the Impact on Food Security in the Near East and North Africa: How to Respond?. (2020). Available online at: 10.4060/ca8430en (accessed June 30, 2020).

[41] Food and Agriculture Organization (FAOSTAT) Crops and Livestock Products. FAO Statistical Databases & Data-Sets. (2020). Available online at: http://www.fao.org/faostat/en/#data/TP (accessed July 5, 2020).

[42] AGRILINKs COVID-19 Impacts on Meat and Dairy Systems in Zimbabwe and Ethiopia. (2020). Available online at: https://www.agrilinks.org/post/covid-19-impacts-meat-and-dairy-systems-zimbabwe-and-ethiopia (accessed June 30, 2020).

[43] EURACITY Germany to Relax Coronavirus Border Controls for Farm Workers. (2020). Available online at: https://www.euractiv.com/section/agriculture-food/news/germany-to-relax-coronavirus-border-controls-forfarm workers/ (accessed July 5, 2020).

[44] Attwood J World's Top Pork Company Closes More Plants in Domino Effect. Blomberg (2020). Available online at: https://www.bloomberg.com/news/articles/2020-04-15/smithfield-foods-to-close-twoadded-meat-processing-facilities (accessed June 28, 2020).

[45] Hein T Covid-19 May Cause Shortage of Labour in NA Pork Sector. Pig Progress (2020). Available online at: https://www.pigprogress.net/World-of-Pigs1/Articles/2020/3/Covid-19-may-cause-shortageof-labour-in-NA-pork-sector-563355E/ (accessed June 28, 2020).

[46] Food and Agriculture Organization (FAO) Mitigating the Impacts of COVID-19 on the Livestock Sector. (2020). Available online at: http://www.fao.org/documents/card/en/c/ca8799en/ (accessed June 28, 2020).

[47] Good K As COVID-19 Slows Meat Processing, Meat Shortages a Growing Concern; Livestock Producers Face Tough Choices. Farm Policy News (2020). Available online at: https://farmpolicynews.illinois.edu/2020/04/as-covid-19-slows-meat-processing-livestock-producersmay-face-tough-choices/ (accessed June 28, 2020).

[48] World Farmers' Organization (WFO) COVID-19 Pandemic Outbreak: Overview of the Impact on the Agricultural Sector. A Technical Assessment of the Undergoing Situation. (2020). Available online at: https://www.wfo-oma.org/wp-content/uploads/2020/05/COVID19-WFO-technical-assessment_005082020.pdf (accessed June 30, 2020).

[49] Congressional Research Service (CRS) Global Economic Effects of COVID-19. (2020). Available online at: https://crsreports.congress.gov R46270

[50] Rossi R European Parliamentary Research Service (EPRS) EU Trade and Transport of Live Animals. (2020). Available online at: https://www.europarl.europa.eu/RegData/etudes/ATAG/2020/646170/EPRS_ATA(2020)646170_EN.pdf (accessed August 5, 2020).

[51] Coronavirus: No Evidence That Food Is a Source or Transmission Route (2020). Available online at: https://www.efsa.europa.eu/en/news/coronavirus-no-evidence-food-source-or-transmission-route (accessed July 9, 2020).

[52] National Cattleman's Beef Association (NCBA) (2020) Available online at: https://www.ncba.org/newsreleases1.aspx?newsid=7225 (accessed July 9, 2020).

[53] Barrett R Wisconsin Farmers Forced to Dump Milk as Coronavirus Slams a Fragile Dairy Economy. Milwaukee Journal Sentinel (2020). Available online at: https://eu.jsonline.com/story/money/2020/04/01/coronavirus-forces-dairy-farmers-dump-milkwisconsin-covid-19/5108609002/

[54] Food Security and Nutrition Quarterly Brief (FSNAU-FEWS NET) (2020). Available online at: https://www.fsnau.org/downloads/FSNAU-Quarterly-Brief-May-2020.pdf (accessed June 30, 2020).

[55] Rossi R European Parliamentary Research Service (EPRS) Protecting the EU Agri-Food Supply Chain in the Face of COVID-19. (2020). Available online at: https://www.europarl.europa.eu/RegData/etudes/BRIE/2020/649360/EPRS_BRI(2020)649360_EN.pdf (accessed August 5, 2020).

[56] Galanakis C. Preface In: Galanakis CM, editor. Proteins: Sustainable Source, Processing and Applications. Waltham, MA: Elsevier Inc (2019) 41–53.

[57] Chriki S Hocquette JF. The myth of cultured meat: a review. Front Nutr. (2020) 7:7. 10.3389/fnut.2020.00007 32118026 PMC7020248

[58] Aleksandrowicz L Green R Joy EJM Smith P Haines A. The impacts of dietary change on greenhousegas emissions, land use, water use, and health: a systematic review. PLoS ONE. (2016) 11:e165797. 10.1371/journal.pone.0165797 27812156 PMC5094759

[59] Hafez HM Attia YA. Challenges to the poultry industry: current perspectives and strategic future after the COVID-19 outbreak. Front Vet Sci. (2020) 7:516. 10.3389/fvets.2020.00516 33005639 PMC7479178

[60] Hashem NM Soltan YA El-Desoky NI Morsy AS Sallam SMA Effects of *Moringa oleifera* extracts and monensin on performance of growing rabbits. Lives Sci. (2019) 228:136–43. 10.1016/j.livsci.2019.08.012

[61] Soltan YA Hashem NM Morsy AS El-Azrak KM Nour El-Din A Sallam SM Comparative effects of *Moringa oleifera* root bark or monensin supplementation on ruminal fermentation, nutrient digestibility and growth performance of growing lambs. Anim Feed Sci Technol. (2018) 235:189–201. 10.1016/j.anifeedsci.2017.11.021

[62] Tait-Burkard C Doeschl-Wilson A McGrew MJ Archibald AL Sang HM Houston RD. Livestock 2.0–genome editing for fitter, healthier, and more productive farmed animals. Genome Biol. (2018) 19:204. 10.1186/s13059-018-1583-1 30477539 PMC6258497

[63] Correddu F Lunesu MF Buffa G Atzori AS Nudda A Battacone G. Can agro-industrial by-products rich in polyphenols be advantageously used in the feeding and nutrition of dairy small ruminants? Animals. (2020) 10:131. 10.3390/ani10010131 31947543 PMC7022336

[64] Makkar HP Smart livestock feeding strategies for harvesting triple gain–the desired outcomes in planet, people and profit dimensions: a developing country perspective. Anim Prod Sci. (2016) 56:519–34. 10.1071/AN15557

[65] Flachowsky G Gruen M Meyer U Feed-efficient ruminant production: opportunities and challenges. J Anim Feed Sci. (2013) 22:177–87. 10.22358/jafs/65962/2013

[66] Wesana J Gellynck X Dora MK Pearce D DeSteur H Measuring food losses in the supply chain through value stream mapping: a case study in the dairy sector. In: Galanakis CM, editor. Saving Food. Chania: Elsevier, Academic Press (2019). p. 249–77. 10.1016/B978-0-12-815357-4.00009-2

[67] Misra NN Dixit Y Al-Mallahi A Bhullar MS Upadhyay R Martynenko A IoT, big data and artificial intelligence in agriculture and food industry, In: IEEE Internet of Things Journal (2020). 10.1109/JIOT.2020.2998584.

[68] Lormore M Managing Milk Production During COVID-19: It's Up for Debate. (2020). Available online at: https://hoards.com/blog-27766-managing-milk-production-during-covid-19-its-up-fordebate.html?fbclid =IwAR0tSNcLFAG0tpDm4HLAWcO8oDfRqGYLwek1owCsWVD2A0zoP8Q90ot7rM (accessed August 3, 2020).

